# Personalized Recommendation Algorithm of Smart Tourism Based on Cross-Media Big Data and Neural Network

**DOI:** 10.1155/2022/9566766

**Published:** 2022-06-26

**Authors:** Jing Lu

**Affiliations:** Department of Management, Zhengzhou University of Technology, Zhengzhou 450000, China

## Abstract

Accurate recommendation of tourist attractions is conducive to improving users' travel efficiency and tourism experience. However, the choice of tourism feature factors and the difference of recommendation algorithm will affect the accuracy of scenic spot recommendation. Aiming at the problems of sparse data, insufficient tourism factors, and low recommendation accuracy in the existing tourism recommendation research, this paper puts forward a scenic spot recommendation method based on microblog data and machine learning by using the characteristics of personalized expression and strong current situation of microblog data and the intelligent prediction function of machine learning, so as to realize accurate and personalized scenic spot recommendation. This paper extracts rich tourism characteristic factors. Typical tourism recommendation algorithms choose tourism characteristic factors from scenic spots, tourists, and other aspects, without considering the travel time, tourism season, and other contextual information of tourists' destination, which can help understand users' tourism preferences from different angles. Aiming at the problem of sparse data and cold start of collaborative filtering recommendation algorithm, this paper introduces deep learning algorithm and combines the proposed multifeature tourism factors to build dynamic scenic spot prediction models (random forest preferred attraction prediction (RFPAP) and neural networks preferred attraction prediction (NNPAP)). The experimental results show that RFPAP and NNPAP methods can overcome the problem of data sparsity and achieve 89.61% and 89.51% accuracy, respectively. RFPAP method is better than NNPAP method and has stronger generalization ability.

## 1. Introduction

Driven by global tourism, supply side reform, and residents' consumption upgrading, the domestic tourism market shows a trend of sustained and rapid growth, and tourism has become an important part of people's leisure life [1]. At present, the tourism information on the Internet is growing at an alarming rate every day. However, in the face of massive tourism information resources, it is difficult for users to choose suitable scenic spots [2].

The key to the accurate recommendation of tourism information lies in how to find tourists' preferences and the design of recommendation algorithm [3]. However, tourist information is often not easy to obtain, so it is difficult to cluster their preferences. With the popularity of social networks such as microblog, the rich user behavior data in the network has brought new opportunities and challenges for accurate tourism information recommendation applications. Compared with other recommended projects, tourism recommendation has its own particularity [4]. The choice of tourist attractions includes not only people's subjective decision-making factors but also contextual factors related to the current situation of users [5]. Literature [6] believes that tourism is the most complex recommended project compared with low complexity projects such as books and films. At present, there are many research works on tourism recommendation algorithms, which can be divided into content-based recommendation algorithm, collaborative filtering recommendation algorithm, and hybrid recommendation algorithm [7–9].

(1) The content-based recommendation algorithm is to establish an interest description for the target scenic spot according to the user's historical tour track and find and recommend scenic spots similar to the target user's interest by establishing the user's interest model or user portrait. Literature [10] designed and developed TripTip recommendation system according to the preference of users to choose scenic spots and recommended similar scenic spots for tourists by analyzing their historical visit locations. Literature [11] used Wikipedia and Twitter to determine the vocabulary related to seasonal tourism, matched the tourist information with the characteristics of scenic spots, and realized the research on scenic spot recommendation of seasonal tourism. Literature [12] recommends scenic spot information according to the seasonal characteristics of scenic spots, scenic spot popularity, and user interest, finds the rules suitable for the personalized recommendation needs of scenic spots, and improves the accuracy of scenic spot recommendation. (2) The recommendation algorithm based on collaborative filtering is to assist in the recommendation of target users according to the historical scores of other users on scenic spots and recommend scenic spots to target users by using the preference data of adjacent users [13]. Literature [14] calculates the user similarity according to the user's historical tour track, measures the user's interest in the scenic spot by using the user's score on the scenic spot, and recommends the scenic spot with high score to similar users. Literature [15] uses the fuzzy c-means method to carry out the recommendation model based on users and items and uses the MAE value to evaluate its accuracy, which can significantly improve the accuracy of collaborative filtering recommendation. Literature [16] designed a tag-based collaborative filtering scenic spot recommendation algorithm. Compared with the collaborative filtering algorithm based on user social relations, its accuracy and coverage are improved by 10% and 4%, respectively, and 15% compared with the project-based collaborative filtering algorithm. It shows that creating tags for scenic spots can effectively improve the accuracy of scenic spot recommendation. Literature [17] makes use of tourists' ratings of tourist attractions to actively select and recommend appropriate tourist attractions for users. (3) Hybrid recommendation algorithm is a method formed by integrating two or more recommendation algorithms. It combines a variety of algorithms to take advantages and avoid disadvantages, so as to achieve better tourism recommendation effect. Literature [18] studies the content-based and context-aware hybrid recommendation system, establishes the knowledge base combined with the information of tourism ontology and scenic spots, and uses the content-based and context-aware information technology to associate with the user's geographical location and filters the recommendation results according to the keyword matching knowledge base, which further improves the accuracy of the recommendation system. Literature [19] designed a hybrid recommendation system based on ontology and collaborative filtering. Semantic integration and cluster analysis of population characteristics, scenic spots, and routes are carried out through ontology. According to user association and modelling, the user's interest and confidence are calculated, which provides a reference scenic spot recommendation list for target users. Literature [20] combines collaborative filtering algorithm with argot semantic model to realize the research on tourism recommendation of ancient villages and scenic spots. The argot semantic model with time weight is used to reduce the dimension of data and improve the accuracy of tourism recommendation. Literature [21] proposes a feature scenario based tourism hybrid recommendation algorithm. Firstly, its preference model is constructed by recording the user behaviour characteristics, and then the scenario model is constructed by using the label recommendation algorithm. Finally, the recommended scenic spots output by the former model are used as the input of the latter model for secondary recommendation. The results show that the accuracy of the hybrid recommendation result is higher than that of a single algorithm. Literature [22] analyzes users' access information to scenic spots and recommends tourism users according to users' geographic information. Literature [23] uses Bayesian network to analyze user data and construct intelligent tourism recommendation system. In recent years, smart tourism recommendation system has developed rapidly in China. It is not only the product of the combination of information technology and tourism resources but also a major change in the concept of tourism development [24]. However, there are still many problems in the smart tourism recommendation system, such as poor performance of the recommendation system and inaccurate recommendation information [25].

To solve the above problems, this paper proposes an intelligent tourism personalized recommendation algorithm based on cross-media big data and neural network. The contributions are summarized as follows: (1) This paper extracts a wealth of tourism characteristic elements, regardless of the travel time, travel season, and other contextual information of the tourist destination, which can help users understand tourism preferences from different angles. (2) Aiming at the problem of data sparsity and cold start of collaborative filtering recommendation algorithm, this paper introduces deep learning algorithm and constructs dynamic scenic spot prediction models (random forest preferred attraction prediction (RFPAP) and neural networks preferred attraction prediction (NNPAP)). (3) This paper designs system architecture design for tourism big data platform and verifies the superiority of the algorithm based on this.

The rest of this paper is organized as follows. The second part discusses research on cross-media tourism big data platform system. In the third part, personalized recommendation algorithm of smart tourism based on cross-media big data and neural network is studied.  Section 4 presents the experiment and result analysis. Finally, the full text is summarized in Section 5.

## 2. Research on Cross-Media Tourism Big Data Platform System

### 2.1. Overall Structure Design of Tourism Big Data

With the rapid development of the Internet and the continuous construction of informatization in China's tourism industry, smart tourism came into being as a technological innovation. Smart tourism mainly obtains information by establishing a cloud platform based on Internet tourism big data, integrates and manages the whole tourism resources based on machine learning and artificial intelligence, and excavates valuable tourism information for tourists and tourism industry. Most of the tourism big data from the Internet are unstructured data.

**Figure 1:**
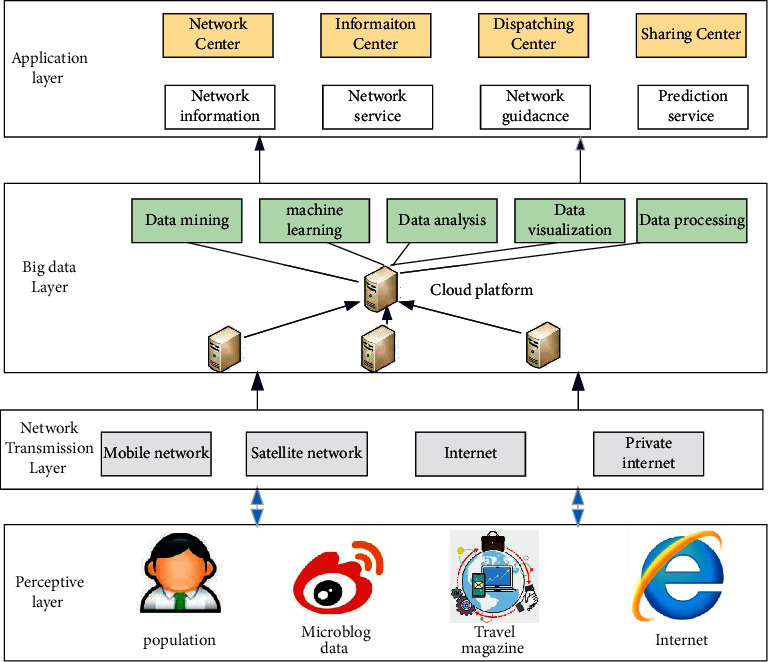
System architecture design for tourism big data platform.

These cross-media data such as text and images from tourism websites or social networks contain rich, unanalyzed, and nonexplicit space-time information and semantic information. They are very important for perceiving the tourism environment and tourist status and providing personalized services on demand. The system architecture for tourism big data platform is designed in Figure 1. As is shown, construction of smart tourism cloud data centre cantered on “coconstruction, sharing, and interconnection.” The smart tourism cloud data centre mainly manages all databases of smart tourism by classification with the help of data warehouse and cloud computing technology. On the basis of data integration management, with the help of cloud computing technology, it provides data information and computing services for various application systems through a shared service platform. The cloud data centre innovates the data collection, transmission, storage, and processing methods, which is the fundamental demand for the construction of smart tourism and the foundation to ensure more intelligent tourism services. Combined with the historical data of tourist flow and the information of tourist origin, we can mine and analyze the consumption patterns and behaviour preferences of different groups of tourists. In order to provide targeted data support for tourism product design and accurate tourism marketing, we need to build a cloud business management platform. On the basis of traditional network marketing, this platform uses data mining technology to cluster analysis and mining association rules for all tourism products. When tourists visit for the first time, make statistical analysis on their consumption characteristics to provide data support for the choice of investment direction of scenic spot tourism market. According to the historical data such as tourists' play records and accommodation information, intelligently analyze their consumption preferences, so as to facilitate the targeted promotion of personalized tourism information for revisited tourists. Tap the potential tourist market with the help of interactive sharing platform. For foreign tourists, recommend local catering and accommodation according to their nationality and personal preferences, and push tourism information in mainstream languages suitable for tourists' nationality. The core design of Hadoop framework is MapReduce and HDFS. MapReduce is a programming model. Programming with its interface can realize the parallel operation of large-scale datasets with multiple machines. It mainly includes map process and reduce process. Its idea is evolved from functional programming method, which provides great convenience for developers.

### 2.2. Design of Cross-Media Tourism Big Data Perception Model Based on Agent

For tourism information, it is necessary to propose a dynamic real-time system that can adapt to various situations, has complexity and scale, and has strong reliability, robustness, and adaptability to Internet environment and external interference. The perception system model of Internet tourism big data is based on the data processing model of Internet tourism information perception system based on cross-media tourism data fusion. From multiple perspectives, the basic element of the cross-media tourism information perception system is the original data of the Internet. It collects and perceives the Internet data, processes and schedules the obtained Internet data, and finally presents the perceived Internet tourism information through the agent-based cross-media tourism big data perception system.

**Figure 2:**
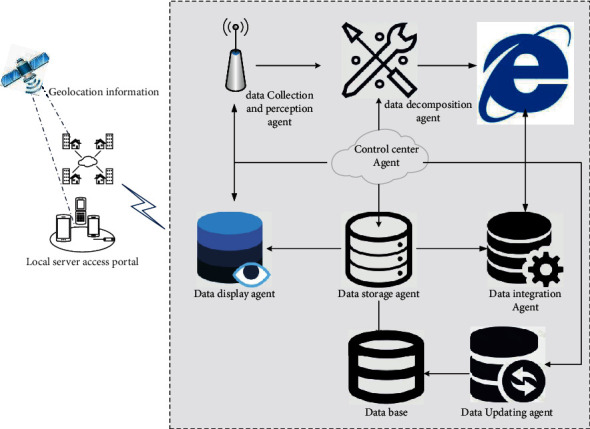
Architecture figure of big data perception model based on agent.

This paper mainly obtains cross-media tourism big data through the Internet and establishes an agent-based cross-media tourism big data perception model. Through multiagent technology, the cross-media tourism big data perception process is encapsulated into relatively independent modules, which are mainly divided into data acquisition agent, data perception agent, data update agent, data decomposition agent, data storage agent, data integration agent, central control agent, data display agent, and other modules, as shown in Figure 2.

1. The data collection agent module is the input module of the whole model. The main function of this module is to collect the cross-media tourism big data of the Internet through the corresponding crawler program, preliminarily select and filter the tourism data, and hand over the data to the data decomposition agent for processing.
2. The data perception agent module is an important module of the whole model. The module determines the perception intensity of the perceived tourism information. For the detailed tourism information, it processes and calculates it according to the web data perception algorithm and weighting rules. Through the calculation of the tourism perception intensity, it selects the valuable tourism data related to tourism and obtains the meaningful information related to tourism; this module involves the perception algorithm based on tourism information in the perception process.
3. The data update agent module is also another important module. The module updates the information collected in the database in real time according to the web page update prediction algorithm, which ensures the timeliness of web page update and avoids the waste of system resources.
4. The data decomposition agent module mainly extracts, filters, and decomposes the data collected by the data collection agent, decomposes the collected web page content into various parts, and finally gives it to the data perception agent for analysis.
5. The data integration agent module is mainly to integrate the data obtained from the data aware agent and hand over the integrated data to the data storage agent for processing.
6. The data storage agent module mainly needs to store the integrated tourism information as the corresponding tourism data through the managed storage of the database and provide it to the data display agent module for use.
7. The data display agent module is the output module of the whole model. The main function of this module is to present the required tourism information to the corresponding service object. The information presentation agent module is directly oriented to the service object. The service object finds the meaningful Internet tourism information they need through the information presentation agent module, and the information presentation agent module also actively interacts the perceived tourism information into its own module through the information storage agent module, and outputs meaningful tourism information through the database for service objects.
8. The central control agent module is the core module of the whole model. The module closely connects the data acquisition agent, data perception agent, data update agent, data decomposition agent, data storage agent, and data integration agent. It is the core organization of the whole perception system and controls the information exchange between various agents. It manages the processed URL queue and pending URL queue established in the process of obtaining Internet tourism data. Through the central control agent module, the whole system can effectively process data and communicate information.

## 3. Personalized Recommendation Algorithm of Smart Tourism Based on Cross-Media Big Data and Neural Network

**Figure 3:**
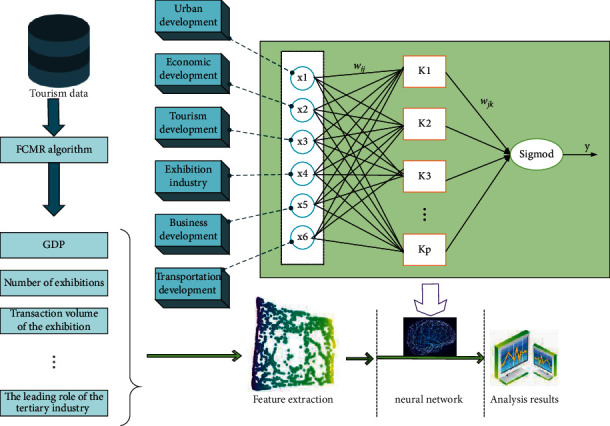
Overall structure figure of algorithm.

Data is the carrier and intelligence is the goal. As a technology and method from data to intelligence, deep learning is the core of data science. Tourism data itself cannot present any valuable information, but useful information can be mined from massive tourism data through in-depth learning. The prediction model of tourist preference scenic spots based on in-depth learning is established. Through experimental comparative analysis, the model with high prediction accuracy is selected as the prediction model of tourist preference scenic spots in this paper. The model structure is shown in Figure 3.

### 3.1. Research on Information Feature Extraction Algorithm Based on Tourism Theme and Temporal and Spatial Characteristics

**Figure 4:**
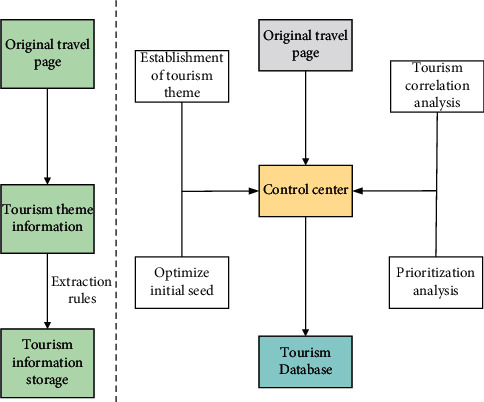
Information acquisition model based on tourism theme and temporal-spatial feature.

The information acquisition based on tourism theme can fully take advantage of the rationality of this trend nature. Starting from the initialization seed URL scheduled at the beginning, combined with the tourism information already obtained, the priority of the access order required by all waiting queues can be calculated. That is, before processing a link, the web pages related to the link and the tourism relevance are calculated and used as the judgment standard to obtain the adjacent web pages oriented to tourism information. The information acquisition model based on tourism theme and temporal-spatial feature is shown in Figure 4.

Firstly, establish a basic tourism keyword thesaurus, and set up appropriate weights in combination with its tourism related level and occurrence frequency. The basic vector of a tourism keyword is as follows:

(1) $$\begin{array}{c} d_{0}=\left(w_{1,0},w_{2,0},\ldots ,w_{n,0}\right). \end{array}$$

The vector formed by the first basic web page *i* is

(2) $$\begin{array}{c} d_{i}=\left(w_{1,i},w_{2,i},\ldots ,w_{n,i}\right). \end{array}$$

In order to avoid the adverse impact of the data information of large text on the calculation of tourism relevance, a tourism web page should include various factors of tourism data, set *w* and the *i*-th relevant factor of QS tourism data, *i* = [l, *n*]. Take enough tourism web pages as test samples and count the times of each factor to determine its weight in measuring the tourism relevance of a web page. Then, the tourism relevance intensity of a web page is

(3) $$\begin{array}{c} V=F1\left(d_{0},d_{l}\right)=\frac{\sum_{j=1}^{j=n}w_{j,0}w_{j,i}}{\sqrt{\sum_{j=1}^{j=n}w_{j,0}^{2}}\sqrt{\sum_{j=1}^{j=n}w_{j,i}^{2}}}. \end{array}$$

According to the first law of geography, everything is related in spatial distribution. The more adjacent things are, the stronger their relevance is. Therefore, kernel density estimation can be used to measure the spatial connection strength between things and reflect the distribution law of things. Kernel density estimation (KDE) mainly estimates the density of point or line pattern with the help of a moving cell (equivalent to a window). KDE is the most widely used nonparametric estimation technology in spatial analysis. It has the advantages of intuitive expression, simple concept, and easy calculation. In definition, let *x*_1_, *x*_2_,… *x*_*n*_ be *n* sample points extracted from the population with distribution density function *f*, and estimate the value of *F* at a certain point *x*, which is usually estimated by Parzen-Rosenblatt kernel:

(4) $$\begin{array}{c} f_{h}\left(X\right)=\frac{1}{nh}\sum_{i=1}^{n}K\left(\frac{X-X_{i}}{h}\right), \end{array}$$

where *h* represents the bandwidth (*h* > 0) and (*X*−*Xi*) is the distance from the estimated point to the sample *Xi*. Among them, the bandwidth *h* has a great influence on the calculation results.

When *h* increases, the change of point density is smoother, but it will mask the structure of density; when *h* decreases, the estimated point density changes abruptly.

### 3.2. Cross-Media Retrieval Method Based on Local Sensitive Hash Algorithm

In order to improve the efficiency of cross-media retrieval, the feasible method is to reduce the proportion of irrelevant content in the dataset. Using local sensitive hash algorithm to map image data to Hamming space, an efficient cross-media retrieval method is proposed, which can significantly improve the proportion of relevant files in the dataset.

**Figure 5:**
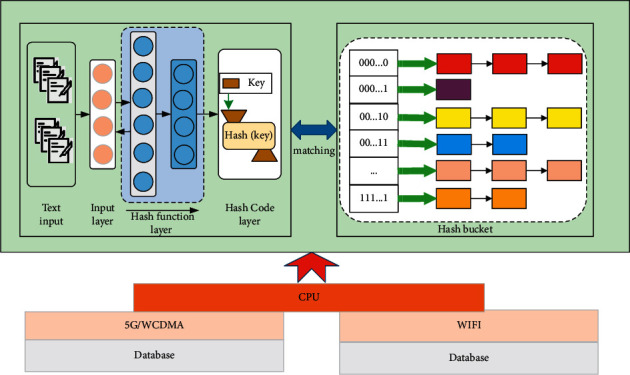
The structure of FCMR algorithm.

Fast cross-media retrieval (FCMR) method includes two stages: local sensitive hash and hash function learning. In the local sensitive hash stage, the local sensitive hash algorithm is used to map the image data to the hash bucket; in the hash function learning stage, neural network learning is used to map the text data to the hash function *h*_*t*_ of the hash bucket. The process of FCMR is shown in Figure 5. To map the image data to *m* hash tables *G*=[*g*_1_, *g*_2_,…*g*_*m*_] ∈ *R*^*k*×*m*^ through local sensitive hash algorithm. In the hash bucket of *m*, where *g* is the set of *m* hash tables, *g*_*j*_ represents the *j*-th hash table, and *k* is the length of the hash code corresponding to the hash bucket. Secondly, the text data is mapped into *m* hash tables through neural network learning. The hash function in its corresponding hash bucket *H*_*t*_=(*Ht*^(1)^, *Ht*^(2)^,…, *Ht*^(*m*)^) represents the learned hash function corresponding to the *j*-th hash table.

Local sensitive hash algorithm is mainly used to solve the approximate nearest neighbour search problem of midpoint in high-dimensional space. The local sensitive hash function is defined as

(5) $$\begin{array}{c} h_{r}\left(p_{i}\right)=\left\{\begin{array}{c} \begin{array}{cc} 1, & r^{T}p_{i}\geq 0 \end{array}\\ \begin{array}{cc} 0, & \text{else} \end{array} \end{array}\right., \end{array}$$

where the hyperplane vector *r* conforms to the Gaussian *n* (0, 1) distribution. Define a series of hash functions *h*_1_, *h*_2_,…, *h*_*n*_, and randomly select *k* of them to form the function *g*(*x*). You may wish to set *h*_1_ to *h*_*k*_; then *g*(*x*)=(*h*_1_(*x*), *h*_2_(*x*),…, *h*_*k*_(*x*)), so as to select *g*(*x*) functions *g*_1_(*x*), *g*_2_(*x*),…, *g*_*m*_(*x*), and each function corresponds to a hash table. Each sample *p*_*i*_ in the image space is mapped to *m* hash tables through *g*(*x*) functions, so that each image sample will appear in a hash bucket of *m* hash tables.

Then the hash bucket corresponding to *p*_*i*_ in the *j*-th hash table can be expressed as

(6) $$\begin{array}{c} g_{j}\left(p_{i}\right)=\langle h_{1}\left(p_{1}\right),h_{2}\left(p_{2}\right),\ldots ,h_{k}\left(p_{i}\right)\rangle . \end{array}$$

When querying, given the query text, *g*(*x*) functions are used to map the query text at the same time, and the image samples falling in the same hash bucket as the query text are taken as the candidate result set. The distance between the query text and the images in the candidate result set is calculated and the accurate retrieval ranking is carried out.

### 3.3. Preference Scenic Spot Recommendation Method Based on Random Forest and Neural Network

**Figure 6:**
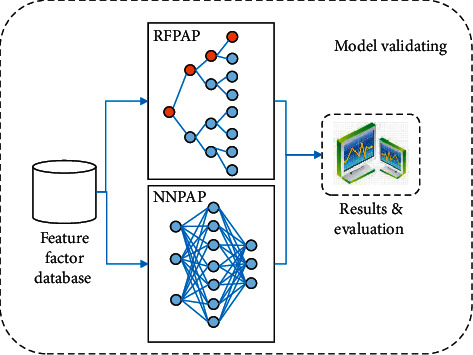
The model flow figure for NNPAP and RFPAP.

Random forest preferred attraction prediction (RFPAP) model and neural networks preferred attraction prediction (NNPAP) model were established by using random forest classification algorithm. The application flow of RFPAP and NNPAP models is shown in Figure 6, including two processes: training and testing. (1) In the training process, input the training data of tourism characteristic factor database, determine the best value of model parameters, and finally construct the RFPAP model. (2) In the testing process, input the test data of the tourism characteristic factor database, apply it to the RFPAP model to predict the preferred scenic spots, obtain the prediction results of the test dataset, compare whether the predicted values are consistent with the actual values, and evaluate the model results.

Random forest is essentially composed of a large number of cart trees. Each decision tree is a classification estimator, which integrates the results of multiple decisions to improve the sensitivity and accuracy of classification. If there are *n* tourists and each tourist has *p* characteristics, and *A* can be formed to *n*×*p* matrix as follows:

(7) $$\begin{array}{c} A=\left[\begin{array}{cccc} a_{1}f_{1} & a_{1}f_{2} & \cdots & a_{1}f_{p}\\ a_{2}f_{1} & a_{2}f_{2} & \cdots & a_{2}f_{p}\\ \vdots & \vdots & \ddots & \vdots \\ a_{n}f_{1} & a_{n}f_{2} & \cdots & a_{n}f_{p} \end{array}\right], \end{array}$$

where *f*_1_, *f*_2_,…, *f*_*p*_ represents the selected *p* characteristic factors and *a*_*ifj*_ represents the measured value of the *j* characteristic factor of the *i*th tourist.

The predicted value is

(8) $$\begin{array}{c} Y=f\left(X\right)=\left\{y_{1},y_{2},\ldots y_{n}\right\}, \end{array}$$

where *f*(·) is a random forest classification function.

Similarly, a model based on neural network can be constructed.

## 4. Experiment and Result Analysis

In data sources, for large-scale standardized tourism websites, the tourism URL is matched through the corresponding regular expression, and the corresponding tourism text and image data in the web page are obtained. For the pages of other websites, there may be no rules to follow, which needs to be judged by the characteristics of tourism data itself. The tour route will include the following: one-day tour, free travel, transportation mode, reservation instructions, route itinerary, and starting and ending points. Take a large number of quality-based tourism web pages as test samples, and set weights according to the frequency of tourism keywords. The important factors affecting the value of tourism information mainly include the name of scenic spots, price, strategy, address, and transportation route. The parameters of neural network mainly depend on training. This paper only needs to design the number of layers of neural network. The neural network structure in this paper is 7-41-6. The activation functions of neural network of input layer, hidden layer, and output layer are sigmoid function, sigmoid function, and softmax function.

In simulation environment, after obtaining 5000 travel web pages of standard travel websites, the experimental configuration environment of this study is as follows: Intel (R) core (TM) i7-6500u, main frequency 2.59 GHz, 16 GB memory, and Windows 10 64-bit operating system. The research experiment of scenic spot prediction is completed by using Sina Weibo tourism dataset and Python scikit-learn library in Spyder integrated development environment.

### 4.1. FCMR Data Retrieval Efficiency Verification

In the experiment, all neural networks are set as three-layer networks, in which the activation function of the output layer is softmax and the activation function of the other layers is sigmoid. Although the network has only three layers, it has achieved satisfactory results.

**Figure 7:**
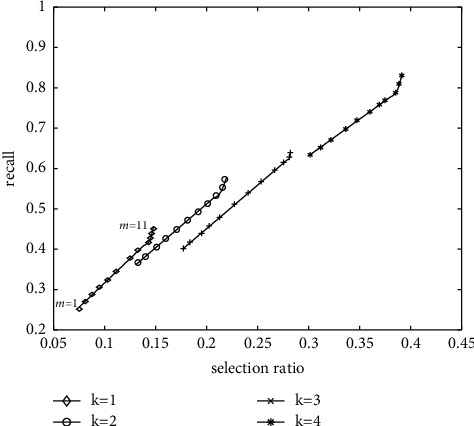
Comparison of text retrieval efficiency of datasets.

Experiments are carried out on the verification set sampled randomly and proportionally in each category on the two datasets to select the parameters *k* and *m* of FCMR model, where *k* is the length of hash code and *m* is the number of hash tables generated in the local sensitive hash stage.  Figure 7 shows the effects of two parameters, hash code length *k* and hash table number *m*, on the model effect from the two directions of text retrieval image and image retrieval text. By observing any graph in Figure 7, it can be found that the influence trend of parameters *k* and *m* on FCMR in two retrieval directions is roughly the same: with the increase of *k*, the recall rate and selectivity gradually decrease. With the increase of *m*, the recall rate and selectivity increase gradually. For different *k*, the change trend of *m* changes. When *k* is 1, the recall rate and selectivity increase in proportion, and the change of growth step is small. When *k* is 5, with the increase of *m*, the growth of recall rate is faster than that of selectivity, and the growth range of both becomes smaller. By comparing the performance of the model on the two datasets, it is found that FCMR has better performance. That is, FCMR has better performance on the retrieval of long text, which also shows that more detailed information is generally required to obtain satisfactory retrieval results to a certain extent.

### 4.2. Evaluation of Prediction Results of Preferred Scenic Spots

**Figure 8:**
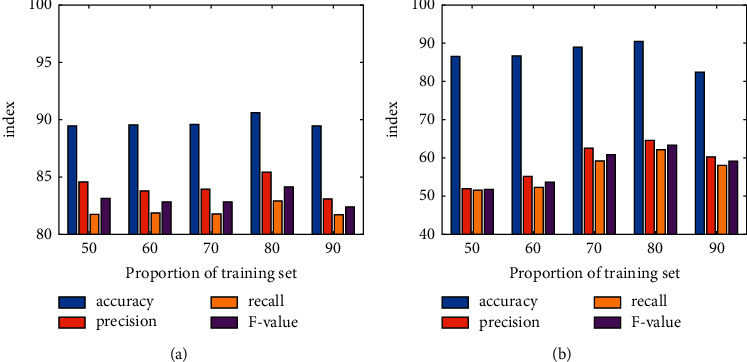
Simulation results and analysis. (a) Prediction accuracy of RFPAP model. (b) Prediction accuracy of NNPAP model.

The optimal segmentation proportion of the model is determined according to the prediction accuracy of the model in different sampling percentages. The prediction results of the RFPAP model are shown in Figure 8. It can be found that when the test set data accounts for 20% of the total dataset, the accuracy rate of the scenic spot prediction results of the RFPAP model reaches the highest value of 89.61%, the accuracy rate is 84.43%, the recall rate is 81.91%, and the *F*-value is 83.15%. When the proportion of the test set data increases or decreases, the accuracy of its evaluation indexes decreases, so it can be determined that this segmentation proportion is the best segmentation proportion of the model. The prediction results of NNPAP model are shown in Figure 8. Compared with other segmentation percentages, when the test set data accounts for 20% of the total dataset, the prediction accuracy rate of the model is 89.51%, the accuracy rate is 62.56%, the recall rate is 60.16%, and the *F*-value is 61.34%, all of which are at the highest value. When the test set data proportion increases or decreases, the accuracy of its evaluation indicators decreases, therefore determining that this segmentation scale is the best segmentation scale of the model.

**Figure 9:**
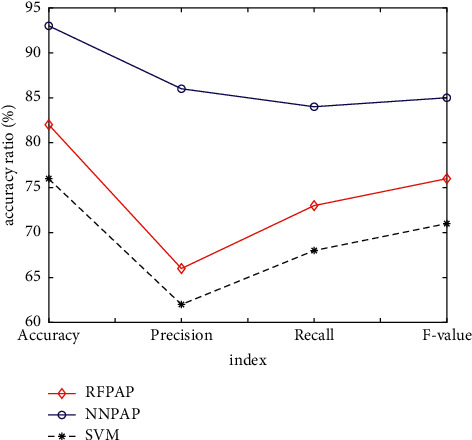
Simulation results comparison.

The application of random forest and neural network algorithm can realize the prediction research of tourist preference scenic spots. In Figure 9, it can be found that the overall prediction effect of RFPAP model is better than that of NNPAP model, and the two have little difference in accuracy. However, compared with the other three indicators, the prediction accuracy of RFPAP model is significantly higher than that of NNPAP model. As shown in Figure 9, the accuracy, recall, and *F*-value of RFP model are 21.87%, 21.75%, and 21.81% higher than those of NNPAP model, respectively. Therefore, RFPAP model is selected as the prediction model of tourist preference scenic spots in this paper.

### 4.3. Comparison Results Analysis

The ability of generalization is the ability to predict unknown data. Discussing the generalization ability of tourist attraction recommendation model is to analyze the applicability of the model proposed in this paper to scenic spots in different regions or different time periods. Because the scenic spot recommendation model RFPAP and the association rule mining itself is to find the regular links between scenic spots from a large database, the generalization ability of the scenic spot recommendation model can be regarded as a measure of the generalization ability of the RFPAP model. In this paper, *k*-fold cross validation is used to verify the generalization ability of RFPAP model. The *k*-fold cross validation is to randomly divide the original sampling dataset into *k* parts and select one part of the data (without repeated extraction) as the test dataset each time, and the other (*k* − 1) parts of the data are used for training, repeat *K* times, and take the average value of the results of *K* times as the final error estimation result. Cross validation can reduce the occurrence of overfitting to a certain extent.

The tourism dataset of Sina Weibo and Python programming language in Spyder integrated development environment are used. In order to more verify the accuracy of the model application, we verify the accuracy and service efficiency of the model over time (6 months, 12 months, and 18 months). The results are shown in Table 1.

Table 1 shows that, with the increase of time, the indexes of the control group did not increase significantly; the experimental group basically kept a balance with the control group in the first six months. After 12 months, various indicators of our recommendation system increased significantly; in particular, the degree of intelligence was greatly improved. The comparison results can verify the reliability and superiority of the scheme.

## 5. Conclusion

In recent years, with the rapid development of web technology and the growth of network resources, people gradually transition from the era of information scarcity to the era of information overload. Facing the explosive growth of tourism information on the Internet, it is difficult for people to quickly obtain tourist attractions that meet their needs and preferences. Tourism recommendation system can effectively solve this problem. However, how to effectively solve the problems of data sparsity and low accuracy of recommendation algorithm is still the focus and difficulty in the research of scenic spot recommendation. This paper studies the prediction model of preferred scenic spots based on random forest and neural network algorithm, uses FP growth algorithm to realize the prediction of related scenic spots, and constructs the scenic spot recommendation model to realize the accurate, intelligent, and personalized recommendation of scenic spots. Firstly, this paper realizes the information acquisition of cross-media tourism big data. For the websites related to tourism information on the Internet, the information of tourism data in combination with the relevance of tourism theme, space-time characteristics, and web page structure characteristics is obtained, and the obtained tourism text and tourism images are displayed. Secondly, based on local sensitive hash algorithm and neural network learning, a new cross-media retrieval method FCMR is proposed, which reduces the retrieval range, improves the proportion of relevant files in the final retrieval range, and then improves the retrieval accuracy; finally, a method of scenic spot recommendation based on random forest is proposed. The construction of scenic spot recommendation model effectively realizes the personalized and accurate recommendation of scenic spots. Generally speaking, tourists will not carry out repeated travel activities; that is, tourists will not visit the same scenic spot many times. However, the scenic spot recommendation method proposed in this paper does not take into account the diversity of scenic spot recommendation, which is likely to lead to the repeated recommendation of the same scenic spot to tourists. Therefore, when establishing the scenic spot recommendation model, the novelty of recommendation needs to be further studied.

## Figures and Tables

**Table 1:** Comparison results analysis.

Indicators	6 months		12 months		18 months	
	Control group	Experimental group	Control group	Experimental group	Control group	Experimental group
Service efficiency	52.63	50.15	60.12	51.25	77.26	83.61
Service evaluation	49.62	48.34	58.64	48.24	79.13	50.78
Prediction accuracy	77.23	75.62	82.61	77.10	92.21	78.95
Intellectualization	36.98	31.65	56.29	33.43	81.27	35.85

## Data Availability

The data used to support the findings of this study are available from the corresponding author upon request.
